# Accounting for the clustering and nesting effects verifies most conclusions. Corrected analysis of: “Randomized nutrient bar supplementation improves exercise-associated changes in plasma metabolome in adolescents and adult family members at cardiometabolic risk”

**DOI:** 10.1371/journal.pone.0275242

**Published:** 2022-10-27

**Authors:** Yasaman Jamshidi-Naeini, Lilian Golzarri-Arroyo, Colby J. Vorland, Andrew W. Brown, Stephanie Dickinson, David B. Allison

**Affiliations:** 1 Department of Epidemiology and Biostatistics, Indiana University School of Public Health-Bloomington, Bloomington, IN, United States of America; 2 Department of Applied Health Science, Indiana University School of Public Health-Bloomington, Bloomington, IN, United States of America; Dartmouth College Geisel School of Medicine, UNITED STATES

## Abstract

In a published randomized controlled trial, household units were randomized to a nutrient bar supplementation group or a control condition, but the non-independence of observations within the same household (i.e., the clustering effect) was not accounted for in the statistical analyses. Therefore, we reanalyzed the data appropriately by adjusting degrees of freedom using the between-within method, and accounting for household units using linear mixed effect models with random intercepts for family units and subjects nested within family units for each reported outcome. Results from this reanalysis showed that ignoring the clustering and nesting effects in the original analyses had resulted in anticonservative (i.e., too small) time x group interaction p-values. Still, majority of the conclusions remained unchanged.

## Introduction

An article by Mietus-Snyder et al. [1] (hereafter “The Article”) reported the results of a randomized controlled trial that examined the effects of nutrient bar supplementation on metabolic biomarkers among adolescents with obesity and their adult caregivers. In The Article, randomization occurred at the level of household units so that adolescents and their caregiver(s) were group randomized to either nutrient bar supplementation or a control condition. However, inferences were drawn from statistical models that ignored the non-independence of observations within the same family unit. Herein, we present a valid statistical model that accounts for clustering and nesting effects within the context of other methodologic choices made by the original authors. We therefore do not intend to address any question or test any hypothesis beyond the scope of The Article.

In The Article, anthropometric and clinical measures were analyzed separately for adolescents and adults. That is, the statistical models with anthropometric and clinical measurements of adolescents as the outcome variable did not include observations from adult participants and vice versa. In the case of plasma ceramides, sphingolipid bases, and amino acids, however, adolescents and adults were analyzed together but the clustering effect of households and the nesting effect that arises from the hierarchical structure of the data were not considered in the statistical analyses. The inconsistency in the analysis of different outcomes aside, we outline below why analyzing households without accounting for the clustering and nesting renders the results and conclusions unverifiable.

When groups (clusters/households), rather than individual subjects, are randomized to experimental conditions, outcome measures of the subjects from the same group are expected to be more similar compared to those from other groups. This within-cluster correlation violates the independent observations assumption, and needs to be accounted for in the statistical analyses [2]. Ignoring the clustering effect, as done in the analyses in The Article, potentially leads to incorrect estimation of the variance and an often-inflated type I error (i.e., anticonservative [that is, too small] p-values) [3, 4]. In The Article, use of incorrect procedures occurs when caregivers and adolescents in the same household are analyzed together as independent observations. Moreover, the control group included two triad family units, one with two adults and one adolescent and the other with one adult and two adolescents. Therefore, even if adolescents and adults are analyzed separately, as reported by The Article for anthropometric and clinical outcomes, there would still be family connections between some subjects in the adult and adolescent subgroups. Therefore, all analyses require accounting for the clustering effect in The Article. A rigorous approach to analyze group randomized trials is including all observations in the statistical model while including a random effect for the cluster. This approach allows for retaining all the information, and accounts for the variability within and among clusters [5].

We attempted to conduct a proper analysis on the data made publicly available as a supplement to The Article. We first attempted to reproduce the original analyses as reported. In doing so, we failed to obtain the same results as those reported in The Article for sphingolipid bases and amino acids. This discrepancy was communicated with the authors of The Article and the journal editors, who acknowledged the errors we had detected. P-values we obtained based on their original analysis approach are reported in our Table 1 below. Additionally, the authors collegially and expeditiously shared additional information on household unit identifiers with us, which is commendable. We also report an updated participant flow diagram in our S1 Fig that corrects the sizes of household units from what is reported in The Article based on the additional information the authors shared with us.

**Table 1:** Time x group interaction p-values from generalized estimating (GEE) and linear mixed effect models (LMM).

Outcome measures	Mietus-Snyder et al. GEE.	Corrected GEE^a^	LMM v1 (without clustering/ nesting)^b^	LMM v2 (with clustering/ nesting)^c^	Comparing LMM v1 to LMM v2; Significance in both, neither, or changed^d^	
					Using p<0.05	Using p≤0.002
	**Fasting plasma ceramides**					
Cer C14:0	0.007	0.007	0.013	0.018	Both	Neither
Cer C16:0	0.024	0.024	0.029	0.035	Both	Neither
Cer C18:0	0.192	0.192	0.229	0.236	Neither	Neither
Cer C18:1	0.058	0.058	0.080	0.088	Neither	Neither
Cer C20:0	0.045	0.045	0.060	**0.068**	**Neither**	Neither
Cer C20:1	0.325	0.259	0.274	0.281	Neither	Neither
Cer C22:0	0.040	0.040	0.055	**0.063**	**Neither**	Neither
Cer C22:1	0.117	0.111	0.125	0.134	Neither	Neither
Cer C24:0	0.356	0.352	0.341	0.348	Neither	Neither
Cer C24:1	0.014	0.014	0.023	0.029	Both	Neither
T. Cer	0.144	0.144	0.147	0.155	Neither	Neither
	**Plasma sphingolipid bases**					
Sphinganine ^e^	p≤0.002 (reported in the text)	0.007	0.013	0.017	Both	Neither
Sphingosine	0.02	0.503	0.573	0.577	Neither	Neither
Dihydro-sphingosine-1-phosphate	0.05	0.488	0.492	0.490	Neither	Neither
Sphingosine -1-phosphate	0.007	0.522	0.549	0.553	Neither	Neither
	**Plasma amino acid metabolites**					
Arginine ^e^	-	0.041	0.065	**0.074**	**Neither**	Neither
Serine	0.0001	0.002	0.006	0.009	Both	**Neither**
Proline	0.0001	<0.001	<0.001	<0.001	Both	Both
Aspartate	0.0001	<0.001	0.001	0.002	Both	**Both**
Cystathionine	0.0001	0.046	0.059	**0.068**	**Neither**	Neither
Sarcosine	0.0001	0.011	0.010	0.014	Both	Neither
Ornithine	0.001	0.178	0.222	0.222	Neither	Neither
Arginine Bioavailability Ratio	0.001	<0.001	0.002	0.003	Both	Changed
Lysine	0.002	0.051	0.061	0.070	Neither	Neither
Alanine	0.003	<0.001	<0.001	0.001	Both	Both
Glutamine	0.007	0.087	0.108	0.115	Neither	Neither
Threonine	0.008	0.032	0.048	0.050	Changed	Neither
Methionine	0.01	0.102	0.144	0.155	Neither	Neither
Fischer ratio	0.01	0.576	0.548	0.537	Neither	Neither
Citrulline	0.02	0.005	0.014	0.019	Both	Neither
Histidine	0.02	0.379	0.354	0.361	Neither	Neither
Tryptophan	0.02	0.405	0.480	0.483	Neither	Neither
Leucine	0.02	0.032	0.064	**0.075**	**Neither**	Neither
Phenylalanine	0.03	0.082	0.115	0.125	Neither	Neither
	**Anthropometric and clinical measures** ^**d**^					
Activity score	-	0.483	0.474	0.479	Neither	Neither
Vitamin D	p<0.05 in both adolescent and adult subgroups	<0.001	0.003	0.005	Both	**Neither**
Weight	-	0.898	0.898	0.898	Neither	Neither
Waist to height ratio	-	0.137	0.162	0.172	Neither	Neither
BMI	-	0.672	0.674	0.677	Neither	Neither
Adiponectin	-	0.551	0.597	0.601	Neither	Neither
HsCRP	-	0.223	0.264	0.271	Neither	Neither
Glucose	-	0.421	0.430	0.435	Neither	Neither
Insulin	-	0.202	0.234	0.242	Neither	Neither
Homeostatic model assessment of insulin resistance	-	0.287	0.310	0.317	Neither	Neither
Plasma total cholesterol	-	0.110	0.134	0.143	Neither	Neither
Plasma triglyceride	-	0.416	0.450	0.455	Neither	Neither
LDL	-	0.222	0.241	0.248	Neither	Neither
HDL	-	0.259	0.302	0.309	Neither	Neither
Systolic blood pressure	p<0.05 in adolescent subgroup	0.058	0.083	0.092	Neither	Neither
Diastolic blood pressure	-	0.235	0.272	0.281	Neither	Neither
Resting heart rate	-	0.106	0.136	0.146	Neither	Neither

^a^ These p-values represent revised values of the incorrect report in The Article after discussions with the authors. These p-values are thus calculated with the model used by Mietus-Snyder et al. that ignores household clustering.

^b^ This model still ignores household clustering and nesting.

^c^ Including random intercepts for household units and adjusted degrees of freedom using between-within method.

^d^ Comparing LMM models with and without clustering and nesting effects being accounted for. We note both <0.05 and ≤0.002 thresholds per the authors’ intention to use them for different outcomes. Because they were used inconsistently in the text, we report here the consequences for both.

^e^ Exact p-values for Arginine, Sphinganine, and anthropometric and clinical measures were not reported in The Article.

To reanalyze the data using procedures that take clustering and nesting effects of households into account, we performed linear mixed effect models (LMM) with random intercepts for family units (to account for the clustering effect) and subjects nested within family units (to account for the repeated measures) on each reported outcome in Tables 2, 4, 5, and 6 of The Article. We used the between-within method to adjust the degrees of freedom (SAS 9.4). The fixed effects in our models were the study group (levels: intervention or control), time (levels: baseline or follow-up), and the interaction between time and group (as the intervention effect). Included covariates were age (in years) and sex (levels: male or female). Our code is available at https://doi.org/10.5281/zenodo.5366705. The raw data we used to generate the results and reach the conclusions in this paper are third party data. Those data (except for household unit identifiers) are publicly accessible from The Article. Household unit identifiers were shared with us by the authors of The Article through personal communications. We did not have any special access privileges that others would not have. To access household unit identifiers, others can contact the corresponding author of The Article and ask them to share household unit identifiers as they did with us. The publicly available dataset did not include participants who had dropped out or those for whom reserved plasma was not available. Therefore, we analyzed available cases only.

The Article described the statistical methods as “a generalized estimating equation [GEE] procedure determined the significance of longitudinal changes […] using age and gender as co-variates”. We outline why we switched from GEE to LMM to reanalyze the data taking clustering and nesting into account. First, The Article reported a study with 11 clusters per intervention and 7 clusters per control (see S1 Fig). GEE is a population-averaged approach [6] using an asymptotic z test that assumes large sample sizes. Thus, GEE should be avoided in the analyses of cluster randomized trials with few clusters [7]. Specifically, GEE based methods use empirical-sandwich estimation for standard errors. When the degrees of freedom are limited, empirical-sandwich estimation leads to unreliable type I error rates in hypothesis testing. That is, when the number of groups per condition is small, the increased variability of the sandwich variance estimator substantially inflates the type I error [8–11]. As stated by Murray et al. “…GEEs may have only limited application in the context of group-randomized trials. The available evidence suggests that they be limited to trials having 20 or more groups allocated to each study condition” [8]. Additionally, there does not appear to be any off-the-shelf software to account for more than two levels of clustering in GEE based models while The Article reported a three-level design (visit, individual subject, household). Thus, it would not be possible to account for the clustering effect of households and the repeated measures using GEE as we did by LMM.

In our Table 1, we present the time x group interaction p-values as reported in The Article using GEE model (which were not reproducible because of discrepancies with the data), our results from corrected GEE model (still ignoring clusters as The Article did), our LMM model that still ignores clustering and nesting (LMM v1: still ignoring clusters, used to compare to LMM v2), and our corrected reanalysis using LMM with adjusted degrees of freedom, family clusters, and repeated measures all being taken into account (LMM v2: a valid approach to clustered data). In The Article, two statistical significance thresholds (<0.05 and ≤0.002) are set for various outcomes. Therefore, in our Table 1, we indicate important differences between the two latter models based on both 0.05 and 0.002 thresholds.

As a result of conducting analyses that account for clustering and nesting effects, we found that ignoring the clustering and nesting effects in the original analysis had resulted in anticonservative time x group interaction p-values, as is well-established in statistical methodological literature. Most time x group interaction p-values increased in our LMM analyses that accounted for nesting and clustering effects, and in the cases just below the statistical significance threshold (i.e., p = 0.05), p-values reported in The Article as statistically significant changed to not statistically significant. Of 13 statistically significant effects at the 0.05 level in LMM model where clustering and nesting effects were ignored (LMM v1), one became non-significant in LMM v2 (Threonine), and of the four statistically significant effects at the 0.002 level in LMM v1, one became non-significant in LMM v2 (Arginine Bioavailability Ratio).

In theory, ignoring clustering yields unbiased estimates of regression coefficients [12], given certain assumptions including that the cluster size is not correlated with cluster-specific treatment effects. Regression coefficients of the LMM and GEE models are presented in our Table 2. Regression coefficients of LMM v1 and LMM v2 are similar for fasting plasma ceramides and plasma sphingolipid bases, and slightly differ for amino acid metabolites. Because our purpose is to provide corrected statistical procedures within the context of methodologic choices of The Article, which involved null hypothesis significance testing based on p-values, we do not elaborate extensively on interpretation of model coefficient estimates.

**Table 2:** Time x group interaction regression coefficient from generalized estimating (GEE) and linear mixed effect models (LMM).

**Outcome measures**	**Corrected GEE** ^**a**^	**LMM v1 (without clustering/nesting)** ^**b**^	**LMM v2 (with clustering/nesting)** ^**c**^
	**Fasting plasma ceramides**		
Cer C14:0	-0.017	-0.017	-0.017
Cer C16:0	-0.023	-0.023	-0.023
Cer C18:0	-0.028	-0.028	-0.028
Cer C18:1	-0.005	-0.005	-0.005
Cer C20:0	-0.256	-0.256	-0.256
Cer C20:1	-0.001	-0.001	-0.001
Cer C22:0	-0.381	-0.381	-0.381
Cer C22:1	-0.004	-0.004	-0.004
Cer C24:0	-0.864	-0.864	-0.864
Cer C24:1	-0.325	-0.325	-0.325
T. Cer	-1.955	-1.955	-1.955
	**Plasma sphingolipid bases**		
Sphinganine	-0.057	-0.057	-0.057
Sphingosine	-0.030	-0.030	-0.030
Dihydro-sphingosine-1-phosphate	-0.001	-0.001	-0.001
Sphingosine -1-phosphate	0.042	0.042	0.042
	**Plasma amino acid metabolites**		
Arginine	17.623	17.802	17.831
Serine	-42.790	-42.044	-42.102
Proline	-75.779	-73.463	-73.439
Aspartate	-13.730	-13.730	-13.671
Cystathionine	-0.216	-0.158	-0.159
Sarcosine	-8.849	-8.849	-8.831
Ornithine	-37.477	-37.364	-37.293
Arginine Bioavailability Ratio	0.449	0.449	0.450
Lysine	-44.646	-43.479	-43.510
Alanine	-84.365	-82.806	-82.774
Glutamine	-73.613	-72.368	-73.116
Threonine	-43.160	-42.244	-42.785
Methionine	-2.551	-2.331	-2.320
Fischer ratio	0.057	0.065	0.067
Citrulline	-45.836	-45.637	-45.660
Histidine	1.297	1.470	1.471
Tryptophan	-3.279	-2.808	-2.823
Leucine	-9.533	-9.013	-8.944
Phenylalanine	-8.408	-8.357	-8.364

^a^ This model represents revised values after discussions with the authors of The Article. These coefficients are thus calculated with GEE model that was used by Mietus-Snyder et al., ignoring clustering and nesting.

^b^ This LMM model does not account for clustering and nesting.

^c^ This LMM model includes random intercepts for household units with adjusted degrees of freedom using between-within method.

Rigor and reproducibility (definitions provided in S1 File) are foundations of scientific advancement, both of which are critical for verification of the results generated using the reported methods. The results reported in The Article were generated using invalid statistical methods for the study design. Our analysis with valid methods produced relatively few differences in dichotomous statistically significant findings. Yet, regardless of the magnitude of difference that valid statistical tests make, the original results are not verifiable (definition provided in S1 File) and cannot be relied upon. Indeed, in similar studies with different numbers of clusters, different numbers of individuals within clusters, or effects closer to the null, the differences in statistical significance may be more or less pronounced. Post-hoc appraisal of how an invalid analysis compares to valid analyses in a particular sample does not justify the original use of the invalid approach.

Although we highlighted changes in statistical significance herein, we recognize that some argue against null hypothesis significance testing using p-values [13]. It is beyond the scope of the current reanalysis activity to discuss the relative value of using p-values or frequentist testing. Rather, we argue that if one chooses to conduct and publish a study that is predicated on frequentist testing and p-values (as the authors of The Article did), one should calculate, use, and interpret p-values correctly. Consequently, changes in statistical significance are simply a dichotomous marker of changes in variance estimates, and thus the same concerns regarding reproducibility and rigor would apply to interpreting confidence intervals because they are based on the same mathematical information.

Our reanalysis has some limitations. First, due to the reasons described in the methods section for switching from GEE to LMM approach, it was not possible to directly compare the findings of our reanalysis that did account for clustering and nesting with the approach used in The Article that ignored those effects. Rather, we needed to conduct the reanalysis using an approach that would allow for clustering and nesting to be accounted for (LMM), but ignore them first (LMM v1). We then compared LMM v1 with the proper analysis that accounted for clustering and nesting (LMM v2). The second limitation is related to the extent we can draw general conclusions about degrees of robustness. We did not conduct simulations or mathematical derivations to show the degrees of robustness on average. Thus, our results only show the degrees of relative robustness of the results and conclusions of this paper (i.e., The Article) when switching from an analysis with incorrect procedures to one with correct procedures. That is, we cannot make any judgement about how often such changes may or may not occur or how large the errors would be. Finally, we did not explore other techniques such as parametric bootstrap to explore how the results would be different compared to LMM. These limitations can be addressed in future research.

We commend Mietus-Snyder et al. for making their raw data publicly available, and their collegiality in providing additional data on family units. In order to be probative, studies need to be rigorously analyzed and transparently reported [14]. Although most conclusions in The Article remain unchanged, ignoring the clustering and nesting effects is an important and common methodological issue in obesity research that leads to unverifiable conclusions [4, 15]. Through our ability to verify and subsequent correcting of the results, we allow them to be used by the readers who might correctly dismiss results and conclusions of The Article because the analyses are conducted using improper statistical procedures for the design. The importance of statistical analysis per the unit of randomization to avoid similar errors in future studies is vital.

## Supporting information

S1 Fig Randomization of family units by Mietus-Snyder et al. (TIF)

S1 File Glossary. Definitions of Reproducibility, Rigor, and Verifiability.
(DOCX)
