# Supplementary figures and images for: Accounting for the clustering and nesting effects verifies most conclusions. Corrected analysis of: “Randomized nutrient bar supplementation improves exercise-associated changes in plasma metabolome in adolescents and adult family members at cardiometabolic risk”
Source: PLoS One. 2022 Oct 27;17(10):e0275242. doi: 10.1371/journal.pone.0275242 (PMC9612448; doi:10.1371/journal.pone.0275242)

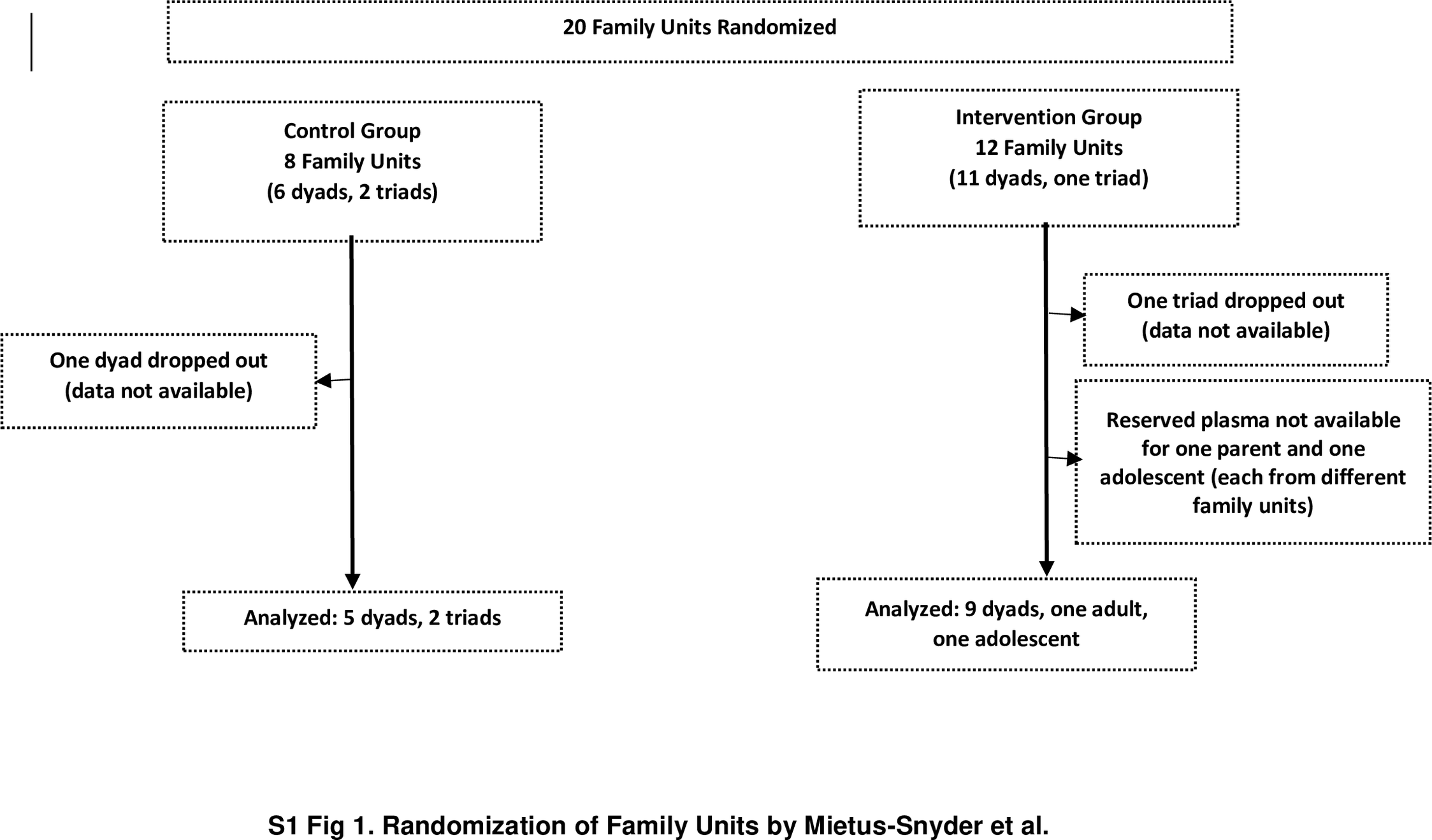

Supplement: S1 Fig — (TIF) [file pone.0275242.s001.tif]
