# Supplementary material for: Accounting for the clustering and nesting effects verifies most conclusions. Corrected analysis of: “Randomized nutrient bar supplementation improves exercise-associated changes in plasma metabolome in adolescents and adult family members at cardiometabolic risk”
Source: PLoS One. 2022 Oct 27;17(10):e0275242. doi: 10.1371/journal.pone.0275242 (PMC9612448; doi:10.1371/journal.pone.0275242)
Supplement: S1 File — Definitions of Reproducibility, Rigor, and Verifiability. (DOCX) [file pone.0275242.s002.docx]

**Glossary***

**Reproducibility:**

The National Academies of Sciences utilizes the following working definition for reproducibility: “Obtaining consistent results using the same input data; computational steps, methods, and code; and conditions of analysis. This definition is synonymous with ‘computational reproducibility’” (National Academies of Sciences, Engineering, and Medicine, 2019, p.46). Disqualifying reproducibility criteria include nonpublic data and code, inadequate record keeping, nontransparent reporting, obsolescence of the digital artifacts, flawed attempts to reproduce others’ research, and barriers in the culture of research (National Academies of Sciences, Engineering, and Medicine, 2019).

**Rigor:**

Scientific rigor is “strict application of the scientific method to ensure robust and unbiased experimental design, methodology, analysis, interpretation and reporting of results” (National Institutes of Health, 2019). Rigor does not guarantee that a study can be reproduced and verified, but conducting a study with rigor makes it more likely.

**Verifiability:**

Verifiability subsumes, but goes beyond, reproducibility. That is, a study is said to be verifiable, and to have been verified, when: (a) the study is reproducible, and the results have been reproduced (by the definition of reproducibility above); and (b) a determination is made that the methods used to generate the results reproduced are valid methods and that the interpretations validly and logically follow from the obtained results.

**References**

National Academies of Sciences, Engineering, and Medicine. (2019). *Reproducibility and Replicability in Science*. Washington, DC: The National Academies Press. Available: <https://doi.org/10.17226/25303>.

National Institutes of Health. 2019. Rigor and Reproducibility in Grant Applications. Available: <https://grants.nih.gov/policy/reproducibility/guidance.htm>

*We have used the exact same definitions as phrased in this glossary in our other currently under review manuscripts or in-press publications (e.g., Jamshidi-Naeini et al. A Practical Decision Tree to Support Editorial Adjudication of Submitted Parallel Cluster Randomized Controlled Trials. *Obesity*. In-press. https://doi.org/10.1002/oby.23373)

.
