# Supplementary material for: Accounting for the clustering and nesting effects verifies most conclusions. Corrected analysis of: “Randomized nutrient bar supplementation improves exercise-associated changes in plasma metabolome in adolescents and adult family members at cardiometabolic risk”
Source: PLoS One. 2022 Oct 27;17(10):e0275242. doi: 10.1371/journal.pone.0275242 (PMC9612448; doi:10.1371/journal.pone.0275242)
Supplement: Attachment — Submitted filename: Response to Reviewers.docx [file pone.0275242.s003.docx]

Dear Dr. Demidenko,

We appreciate your insightful comments on our manuscript. Our replies are in red below.

We noted a theme of incertitude about our scientific contribution and the scope of the current re-analysis activity. Thus, we provide some clarification herein and in the revised manuscript (the version with track changes) (lines 36-40).

Current manuscript is a re-analysis of an existing published study (i.e., referred to in the manuscript as ‘The Article’). Therefore, details regarding the significance, procedures, conduct, and conclusions of the study are provided in The Article. Our concern in the current manuscript is invalidity of the statistical analyses used for generating the published results. We present a valid statistical model and the corresponding results. We do not intend to address any research question or test any hypothesis beyond the results presented in The Article. Journals often handle this type of re-analysis as ‘correspondence’ so that it is linked to the previously published work. Because PLoS ONE editorial policy does not provide such an option, we were advised to submit our re-analysis as an original research article. Thus, our scientific contribution should be evaluated within the context of post-publication re-analysis and error correction.

- The most important results of statistical analysis are the coefficients of the model; the p-values have secondary importance. Therefore, I suggest reporting the coefficients in Table 1, along with the p-values.
- Although an overseen clustering may produce a lower level of statistical significance (larger standard errors and p-values) it yields unbiased coefficients (Demidenko, E. Mixed Models. Theory and Applications with R, Hoboken, NJ: Wiley, 2013).

The well-known consequences of ignoring clustering and nesting effects in the regression models for multilevel data are underestimated standard errors (and p-values) and unbiased regression coefficients [1, 2]. However, debate about the relative value of using p-values or frequentist testing, and whether regression coefficients or the p-values are superior in importance to one another is beyond the scope of this current reanalysis activity. Rather, the authors and the journal have chosen to publish a paper that was predicated on frequentist testing and p-values. Therefore, the testing, calculation, use, and interpretation of p-values should be conducted correctly, but in The Article they were not.

We present the model coefficients in supplementary Table 1.

- Moreover, while the original paper uses GEE you employ LMM. Consequently, the results on p-values must be different.

Thanks for this insightful comment. In lines 97-111 of the manuscript, we outline the reasons why LMM is the legitimate approach to correctly account for clustering and nesting effects given the study design by Dr. Mietus-Snyder et al. Because our purpose is to focus on accounting for vs. ignoring clustering and nesting, we updated our analyses and Table 1 to compare LMMs with and without clustering and nesting effects. Please see Table 1 and lines 97-111.

- Indeed, as follows from Table 1 the proportion of the changed p-values (rows) is only about 5:30. In spite the correct claim of the effect of ignored clustering the results look pale.

Rigor^1^ and reproducibility^2^ are foundations of science advancement, both being critical for verification of the results generated using the reported methods. The results reported by Mietus-Snyder et al. are not generated using valid statistical methods for the study design (our reasoning outlined in the manuscript and elsewhere by our group and others [3, 4]). Thus, regardless of the magnitude of difference that valid statistical tests would make, those results are not verifiable^3^ (please see lines 130-135 too). In this regard, we highlight the following matters:

i. Editors, journals, authors, and the scientific community overall should have a commitment to accuracy and integrity of the science [5], even if the results only change modestly as a result of doing correct analyses.
ii. Hearkening back to our argument about the choice of frequentist testing, correcting the analyses changes qualitative conclusions by the standard operating procedures and social norms of the use of frequentist testing and readers have a right to know this.
iii. Finally, for an astute reader, especially someone writing a blue-ribbon panel report that might utilize such a paper, or a meta-analyst, without seeing the critique and re-analysis results, they will correctly discern that the original results cannot be relied upon because the analyses are invalid. They might therefore be unable to use the study and its findings, even though the study and its findings (once correctly analyzed) do have value. That is, an unverified result is a result that cannot be utilized and relied upon fairly. By our verifying (and correcting) the results, we allow them to be used by people who correctly understand that the current analyses are not correct.

Additionally, and as a side note, the proportion of the changed p-values is essentially a function of what one takes as the denominator and the significance level. As mentioned in the manuscript, we describe this proportion as “of 13 statistically significant effects at the 0.05 level in LMM models where clustering and nesting effects were ignored, one became non-significant, and of the four statistically significant effects at the 0.002 level, one became non-significant”.

^1^Scientific rigor: “the strict application of the scientific method to ensure unbiased and well-controlled experimental design, methodology, analysis, interpretation and reporting of results.” (https://grants.nih.gov/policy/reproducibility/index.htm).

^2^Reproducibility: “An article is designated as reproducible if the [independent investigator] succeeds in executing the code on the data provided and produces results matching those that the authors claim are reproducible” [6].

^3^Verifiability: a study is said to be verifiable, and to have been verified, when: (a) the study is reproducible, and the results have been reproduced (by the definition of reproducibility above); and (b) a determination is made that the methods used to generate the results reproduced are valid methods.

- Some technical description of the statistical model (dependent, independent variables, equations) and how exactly the clustering has been implemented is missing. Therefore, it is impossible to evaluate the improvements and your contribution.

The technical description of our statistical model is presented in the manuscript lines 75-89. We have deposited our statistical code at <https://doi.org/10.5281/zenodo.5366705>.

**References**

1.	Maas CJM, Hox JJ. The influence of violations of assumptions on multilevel parameter estimates and their standard errors. Computational Statistics & Data Analysis. 2004;46(3):427-40. doi: <https://doi.org/10.1016/j.csda.2003.08.006>.

2.	Ntani G, Inskip H, Osmond C, Coggon D. Consequences of ignoring clustering in linear regression. BMC Medical Research Methodology. 2021;21(1):139. doi: 10.1186/s12874-021-01333-7.

3.	Brown AW, Li P, Bohan Brown MM, Kaiser KA, Keith SW, Oakes JM, et al. Best (but oft-forgotten) practices: designing, analyzing, and reporting cluster randomized controlled trials. The American Journal of Clinical Nutrition. 2015;102(2):241-8. doi: 10.3945/ajcn.114.105072.

4.	Murray DM, Varnell SP, Blitstein JL. Design and Analysis of Group-Randomized Trials: A Review of Recent Methodological Developments. American Journal of Public Health. 2004;94(3):423-32. doi: 10.2105/AJPH.94.3.423.

5.	ICMJE. Recommendations for the Conduct, Reporting, Editing, and Publication of Scholarly Work in Medical Journals 2019. Available from: <http://www.icmje.org/icmje-recommendations.pdf>.

6.	Peng RD. Reproducible research and Biostatistics. Biostatistics. 2009;10(3):405-8. doi: 10.1093/biostatistics/kxp014.
