# Supplementary material for: Accounting for the clustering and nesting effects verifies most conclusions. Corrected analysis of: “Randomized nutrient bar supplementation improves exercise-associated changes in plasma metabolome in adolescents and adult family members at cardiometabolic risk”
Source: PLoS One. 2022 Oct 27;17(10):e0275242. doi: 10.1371/journal.pone.0275242 (PMC9612448; doi:10.1371/journal.pone.0275242)
Supplement: Attachment — Submitted filename: Response to Reviewers.docx [file pone.0275242.s004.docx]

Dear Dr. Demidenko,

Thank you for your insightful comments, and giving us the opportunity to improve the manuscript. Our replies are in blue below.

1. It should be mentioned in the paper that according to the theory (Demidenko, 2013) accounting for family clustering should not affect the coefficients but increase the standard errors and reduce the p-values. The current study confirms this expectation.
2. I suggested reporting the coefficients themselves as well, but they are missing in the current version, again.

Thank you for your cogent comment. We agree that as stated in (Demidenko, 2013), [under certain assumptions, including that the cluster size is not correlated with cluster-specific treatment effects], ignoring within-cluster correlation yields unbiased estimates of regression coefficients ((1), pages 42 and 157), but artificially reduced (more significant) standard errors and p-values (2). Though, of course, they could change in any one sample. Similarly, there appears to be little bias (less than ±10%) in the estimates of regression coefficients across the methods that accommodate clustered data for 10 or fewer clusters with few (e.g., 7-14) observations per cluster (3, 4).

We have now included coefficients in our manuscript per your suggestion (see Table 2 and lines 139-146). As we report in the manuscript, “regression coefficients of LMM v1 and LMM v2 are similar for fasting plasma ceramides and plasma sphingolipid bases, but slightly differ (3% or less) for amino acid metabolites.” However, because our purpose is to provide corrected statistical procedures within the context of methodologic choices of The Article (i.e., null hypothesis significance testing using p-values), we do not dwell extensively on the interpretation of regression coefficient estimates.

3. The p-values from GEE and LME are not compatible because the underlying statistical models are different. Thus, we must compare LMM v1 versus LMM v2 to understand the effect of family clustering. As follows, the difference in p-values is negligible.

Thank you for these remarks. We agree that the comparison must be between the results derived from similar approach. Owing to your insightful comments on the previous version of our manuscript, we included LMM v1 and we are comparing LMM v2 vs. LMM v1 both in Table 1 and to summarize our findings. We also agree that the model that ignores clustering (LMM v1) yields roughly the same answers as the model that accounts for clustering and nesting (LMM v2). As we outline in the manuscript, we believe regardless of the magnitude of difference that valid statistical tests would make, results that are derived from inappropriate procedures for the design (which is the case for either GEE or LMM v1) are unsubstantiated and not verifiable. That is, incorrect analysis sometimes yields the correct answers and correct analysis sometimes yields incorrect answers (due to type I and/or type II errors). The fact that LMM v1 yields roughly the same answer as LMM v2 does not mean that ignoring clustering (and nesting) is a negligible issue. Our reanalysis of this article now allows the scientific community to interpret the results in the context of an analysis strategy appropriate to the study design, increasing the confidence in its findings.

4. Graphic presentation where x-axis is time would be very beneficial to see the differences between GEE, LMM v1, and LMM v2 models in addition to Table 1.

Thank you for this comment. We emphasize that our manuscript is not intended to be a methods paper. We are making a critique of an existing published paper because the results of that paper are produced using inappropriate procedures for its design. While we argue that the results are unsubstantiated, we cannot show that they are wrong or right because doing so would require knowing the *‘true’* answer which would be possible by conducting simulations or mathematical derivations to show the degrees of robustness. We propose that keeping the values in our table format is optimal to present our reanalysis, as the original authors did in their paper.

5. Due to my previous point paper’s contribution is marginal. Yes, there is a slight improvement, but it is wrong to say that the analysis of “The Article” is incorrect. I suggest the language “adjustment” not saying that the previous analysis is wrong. This statement must be a part of the study limitations. Is it true that n=14 as follows from S1 Fig 1?

- Thank you for raising this point. We agree that our writing has not been as clear as it could have been. Our statements about the ‘invalid’ analysis in The Article could be interpreted in two ways: 1) that the procedures used to produce the findings were incorrect for the purported properties of the study design, 2) that the results produced by the procedures used are not the results which would be produced by proper procedures. We clarify that we mean the former, not the latter: when we say the analysis is either wrong or invalid, we mean the procedures used to produce the results were incorrect, and when we say results, we mean the findings produced by the procedures used. In this case, the findings from the incorrect procedures and proper procedures are similar. We now have clarified this in our writing.
- We also agree that it is wise to include a study limitations section. Thank you for that suggestion. We now have included the following study limitations section to the manuscript:

“*Our reanalysis has some limitations. First, due to the reasons described in the methods section for switching from GEE to LMM approach, it was not possible to directly compare the findings of our reanalysis that did account for clustering and nesting with the approach used in The Article that ignored those effects. Rather, we needed to conduct the reanalysis using an approach that would allow for clustering and nesting to be accounted for (LMM), but ignore them first (LMM v1). We then compared LMM v1 with the proper analysis that accounted for clustering and nesting (LMM v2). The second limitation is related to the extent we can draw general conclusions about degrees of robustness. We did not conduct simulations or mathematical derivations to show the degrees of robustness on average. Thus, our results only show the degrees of relative robustness of the results and conclusions of this paper (i.e., The Article) when switching from an analysis with incorrect procedures to one with correct procedures. That is, we cannot make any judgement about how often such changes may or may not occur or how large the errors would be. Finally, we did not explore other techniques such as parametric bootstrap to explore how the results would be different compared to LMM. These limitations can be addressed in future research.*”

- According to the information shared with us by the authors, 20 clusters (i.e., family units) were randomized, among which two families dropped out, and 18 clusters (n=36) were included in the analysis.

**20 Family Units Randomized**

**Intervention Group**

**12 Family Units**

**(11 dyads, one triad)**

**Control Group**

**8 Family Units**

**(6 dyads, 2 triads)**

**One dyad dropped out**

**(data not available)**

**Analyzed: 5 dyads, 2 triads**

**One triad dropped out**

**(data not available)**

**Reserved plasma not available for one parent and one adolescent (each from different family units)**

**Analyzed: 9 dyads, one adult,**

**one adolescent**

**S1 Fig 1.** **Randomization of Family Units by Mietus-Snyder et al.**

1.	Demidenko E. Mixed models: theory and applications with R: John Wiley & Sons; 2013.

2.	Johnson JL, Kreidler SM, Catellier DJ, Murray DM, Muller KE, Glueck DH. Recommendations for choosing an analysis method that controls Type I error for unbalanced cluster sample designs with Gaussian outcomes. Statistics in medicine. 2015;34(27):3531-45.

3.	McNeish D, Stapleton LM. Modeling Clustered Data with Very Few Clusters. Multivariate Behavioral Research. 2016;51(4):495-518.

4.	Maas CJM, Hox JJ. The influence of violations of assumptions on multilevel parameter estimates and their standard errors. Computational Statistics & Data Analysis. 2004;46(3):427-40.
