# Supplementary material for: Accounting for the clustering and nesting effects verifies most conclusions. Corrected analysis of: “Randomized nutrient bar supplementation improves exercise-associated changes in plasma metabolome in adolescents and adult family members at cardiometabolic risk”
Source: PLoS One. 2022 Oct 27;17(10):e0275242. doi: 10.1371/journal.pone.0275242 (PMC9612448; doi:10.1371/journal.pone.0275242)
Supplement: Attachment — Submitted filename: Response to Reviewers.docx [file pone.0275242.s005.docx]

Dear Dr. Demidenko,

Thank you for your insightful comments, and giving us the opportunity to improve the manuscript. Our replies are in blue below.

**Response to the Editor:**

I agree with the reviewer that incorporation of the family cluster effect does not change major conclusions. Consequently, I suggest the authors retitle the paper as follows: "Ignoring the clustering effect does not change major conclusions..." I understand that the authors may disagree with my decision and submit the paper to another journal. As you know, PLOS ONE is among a few scientific journals that publish "negative results" to avoid publication bias. Although adjusting for random effect is the right way to analyze the data it just happened that for this particular study mixed model did not make considerable difference. I encourage the authors to address the comments of the reviewer and resubmit the paper.

Thank you for these comments. We note that the fact that “[…] it just happened that for this particular study mixed model did not make considerable difference” is only known after we have reanalyzed the data using correct statistical procedures. That is, the results and conclusions published by Mietus-Snyder et al. are unsubstantiated because they are drawn from incorrect statistical methods. As a result of our reanalysis, results and conclusions derived from the original GEE models are verified or corrected. We have addressed the point about the title in our responses to reviewer 1.

**Response to Reviewer 1:**

1. I could not find any mention of the conclusions that were changed, only small changes in a minority of p-values. It is stated in the final paragraph that “most conclusions in The Article remain unchanged”. Can the authors explain exactly which study conclusions are changed by clustered analyses and if any, what the significance of these changes to the article’s interpretation? If none, the title might more appropriately read “Incorporation of the clustering effect in group randomized trial does not change the main study conclusions”. It is interesting that the reference 5 provided as an example of use of this clustered approach from the Allison group (Golzarri-Arroyo et al, 2020) similarly did not change study conclusions, but stated that accurately in the title.

Thank you for raising the point about the title. We agree that a change in the title would present the consequences of accounting for clustering and nesting effects in this particular sample more clearly. We changed the title to “Accounting for the clustering and nesting effects verifies majority of conclusions. Corrected analysis of: “Randomized nutrient bar …””. The suggested title by the reviewer “Ignoring the clustering effect does not change major conclusions..." implies that we are encouraging researchers to ignore clustering (and nesting) in cluster randomized trials, which is not our intended message.

We state in our manuscript that most conclusions remain unchanged when the comparison is between linear mixed effects models (LMM) that account for clustering and nesting and LMMs that ignore clustering and nesting. We precisely state variables for which p-values are changed from statistically significant in the incorrect analysis to insignificant when clustering and nesting are accounted for (Threonine and Arginine Bioavailability Ratio). The changes we made to the title will further address this matter. On the other hand, addressing the consequence of accounting for clustering and nesting on the *article*’s conclusions and interpretations should occur under the assumption that the *article*’s conclusions and interpretations are based on reproducible results, but they are not. Such comparison would require re-writing the *article*’s interpretations based on a) “corrected GEE” models and b) consistent specification of the significance level (i.e., 0.002 or 0.05). This is, as correctly mentioned by the reviewer, a “responsibility of the original study team”.

- Both of these PLOS ONE studies the Allison group has reanalyzed incorporating the clustering effect (Mietus-Snyder et al and Short et al) involved minority adolescent populations, who would be at heightened risk for socioeconomic disadvantage. Unfortunately, an important issue relevant to health disparity that a statistical manipulation alone cannot address, is fragmentation of traditional family units and potential breakdown in the predictability of household factors.

Thank you for raising this important matter. We agree that socioeconomic disadvantage and health disparities among adolescences have critical importance. We agree that our reanalysis efforts do not address health disparity issues among adolescents. We do not intend to address any research question with that regard in our manuscript. In this manuscript we are making a critique of an existing published paper because the results of that paper are produced using inappropriate procedures for its design.

2. Can the authors delineate which household factors are felt to be most critical in justification of the proposed clustered analysis? If dietary factors pertain, does clustered analysis reveal any difference in the diets that were reported to be uniformly poor for both adolescents and adults in the original study? If genetic or epigenetic factors are implicated, what effects do nonbiological family units or shared custody have on clustered analyses? These issues raise an important point of discussion that might be expanded upon by the authors to help explain why the application of clustered analysis – at least in this study and the study reanalyzed in reference (5) does not appear to change the main study conclusions reached without clustering.

Thank you for raising these interesting matters. These questions present the potential to expand current knowledge and understanding of the factors that may explain correlation of observations within household units in cluster randomized trials. From a methods aspect, the effect of ignoring clustering would be more moderate in designs with more clusters and fewer individual participants per cluster compared to designs with fewer clusters and larger number of individuals per cluster. Situations in which families are randomly assigned to treatments are examples of smaller cluster sizes, such as the article under question here and the study we referred to in Golzarri-Arroyo et al. Any subjective explanation about dietary or genetic factors and their potential role in similarities or dissimilarities within or among households in this particular sample would be speculation, and not addressable within the context of our reanalysis.

- There are two sets of changes reported upon in this Correction – the first set due to an error in the original time-by-group p-values reported that has apparently been acknowledged by the study authors. It is not apparent why those p-value corrections have not already been published as a Correction as this should be the responsibility of the original study team, once alerted to their error. The title of this Correction by Jamshidi-Naeini implies that it is the application of clustered analysis, not these time x group interaction p-value corrections that changes some conclusions. It does seem apparent that the corrected time by group p-values do not change study conclusions (with the exception of aromatic amino acids tryptophan and phenylalanine, the latter still showing a trend with p = 0.07). This is not however a central observation and does not impact the article’s interpretation.

It is the second set of p-value changes that are contingent on the newly applied clustered analysis that would still be germane to the Correction submitted by Jamshidi-Naeini et al. The authors support their claim that this approach overcomes an important methodological issue in obesity research with several recent reviews on this topic. Their point is well taken, but the frequent use the terms “correctly” and “appropriately” in describing the application of a clustered analysis seems unnecessarily pejorative, since as already noted, the reanalysis has not actually been demonstrated to change the study’s conclusions. It of course changed p-values, as would be expected with a decrease in the number of independent observations, but only a small number of p-values that were nominally significant become slightly insignificant. Trends remained trends and the strongest observations, on which the original study conclusions are based, remain very significant.

Thank you for your cogent comment. 1) We agree that it is the responsibility of the original authors and the journal to correct the irreproducibility issue of the article. Both have been notified that a portion of the analyses in the article are not reproducible. We cannot comment on the reason why these errors have not been publicly acknowledged yet. 2) Our altered title should address the issue raised about the title. 3) The purpose of our manuscript is making a critique of an existing published paper because the results of that paper are produced using inappropriate procedures for its design. We have been factual throughout: majority of conclusions that are derived from correct procedures are the same as those derived from incorrect procedures. 4) Until appropriate corrections are made to the article, any comparison of our results with the article’s interpretations would require specification of which model (LMM or GEE) one is comparing our results with and which significance level (0.05 or 0.002) they are using. 5) We understand that referring to our analysis as a ‘correct’ or ‘appropriate’ analysis may be disapproved by some. We thus modified the language where our message was still clear without those words.

- A 2019 paper cited (ref 6) for which DB Allison is also senior author on “The Need for Greater Rigor in Childhood Nutrition and Obesity Research” states what Jamshidi-Naeini et al were sure to observe in that (quoting from ref 6) “Clustering reduces power”… going on to say “there are instances in which misanalysis of clustered childhood obesity interventions has led to unsubstantiated conclusions.” This certainly is true, but the authors have not demonstrated that it is true in this instance – which circles back to the first two questions above. Which study conclusions have actually been altered by the clustered analysis? And if none, why not?

Thank you for these remarks. There should be a distinction between “unsubstantiated” results vs. “incorrect” results. There are two approaches to consider this matter: 1) that the procedures used to produce the findings were incorrect for the purported properties of the study design, 2) that the results produced by the procedures used are not the results which would be produced by proper procedures. We clarify that we mean the former, not the latter: when we say the analysis is either wrong or invalid, we mean the procedures used to produce the results were incorrect, and when we say results, we mean the findings produced by the procedures used. In this case, the findings from the incorrect procedures and proper procedures are similar.

Results and conclusions of the article are unsubstantiated (i.e., unsupported) without our reanalysis. That is, regardless of the magnitude of difference that valid statistical tests would make, results that are derived from inappropriate procedures for the design are unsubstantiated and not verifiable. Incorrect analysis sometimes yields the correct answers and correct analysis sometimes yields incorrect answers (type I and/or type II errors). While we argue that the results are unsubstantiated, we cannot show that they are wrong or right because doing so would require knowing the ‘true’ answer which would be possible by conducting simulations or mathematical derivations to show the degrees of robustness.

3. A full reading of the original paper reveals a detailed baseline principal components analysis to reduce the dimensionality of the complex lipidomic data set incorporated in baseline correlograms with traditional cardiometabolic risk factors and lipoprotein subspecies. There are also change correlation analyses that reveal numerous hypothesis-generating associations independent of pre-post metabolite p-values. Much of the discussion in the original study is rooted in the correlograms that summarize this body of data. Would clustered analysis lend any further insight to the principal component and correlation analyses? Can the authors explain why their reanalysis does not address these two prominent elements of the original paper?

We did not intend to reanalyze every analysis in the article, and do not claim as such in our manuscript. Principal components analysis is out of the scope of our discussion in this manuscript.

**Response to Reviewer 2:**

The current investigators pointed out a possible design flaw in the manuscript. That is to say “In a published randomized controlled trial, household units were randomized to a nutrient bar supplementation group or a control condition, but the non-independence of observations within the same household (i.e., the clustering effect) was not accounted for in the statistical analyses." Assuming this to be the case, the investigators provided a side by side comparison of results (clustering vs. non clustering) and proceeded accordingly. The results are very similar. However, that would have to be debated with the original investigators and not this reviewer. The work was thorough and certainly appeared to be presented reasonably in a descriptive, and not methodologic, fashion as was pointed out by the investigators.

Thank you for reviewing our manuscript. We notified the original research team and the journal of the issues we detected.
